# Assessing the Risk of Heart Attack: A Bayesian Kernel Machine Regression Analysis of Heavy Metal Mixtures

**DOI:** 10.21203/rs.3.rs-4456611/v1

**Published:** 2024-06-18

**Authors:** Boubakari Ibrahimou, Kazi Tanvir Hasan, Shelbie Burchfield, Hamisu Salihu, Yiliang Zhu, Getachew Dagne, Mario De La Rosa, Assefa Melesse, Roberto Lucchini, Zoran Bursac

**Affiliations:** Florida International University, Robert Stempel College of Public Health & Social Work, Department of Biostatistics; Florida International University, Robert Stempel College of Public Health & Social Work, Department of Biostatistics; Florida International University, Robert Stempel College of Public Health & Social Work, Department of Biostatistics; Baylor College of Medicine, Center of Excellence in Health Equity, Training and Research; University of New Mexico, Clinical and Translational Science Center; University of South Florida, College of Public Health; Florida International University, Robert Stempel College of Public Health & Social Work, Center for Research on US Latino HIV/AIDS and Drug Abuse; Florida International University, College of Arts, Sciences & Education, Department of Earth and Environment; Florida International University, Robert Stempel College of Public Health & Social Work, Department of Environmental Health Sciences; Florida International University, Robert Stempel College of Public Health & Social Work, Department of Biostatistics

**Keywords:** Bayesian kernel machine regression, Heavy metal mixtures, Cardiovascular disease, Heart attack, NHANES

## Abstract

**Background:**

The assessment of heavy metals’ effects on human health is frequently limited to investigating one metal or a group of related metals. The effect of heavy metals mixture on heart attack is unknown.

**Methods:**

This study applied the Bayesian kernel machine regression model (BKMR) to the 2011–2016 National Health and Nutrition Examination Survey (NHANES) data to investigate the association between heavy metal mixture exposure with heart attack. 2972 participants over the age of 20 were included in the study.

**Results:**

Results indicate that heart attack patients have higher levels of cadmium and lead in the blood and cadmium, cobalt, and tin in the urine, while having lower levels of mercury, manganese, and selenium in the blood and manganese, barium, tungsten, and strontium in the urine. The estimated risk of heart attack showed a negative association of 0.0030 units when all the metals were at their 25^th^ percentile compared to their 50^th^ percentile and a positive association of 0.0285 units when all the metals were at their 75^th^ percentile compared to their 50^th^ percentile. The results suggest that heavy metal exposure, especially cadmium and lead, may increase the risk of heart attacks.

**Conclusions:**

This study suggests a possible association between heavy metal mixture exposure and heart attack and, additionally, demonstrates how the BKMR model can be used to investigate new combinations of exposures in future studies.

## Introduction

Cardiovascular disease (CVD) is a serious global health issue. Despite recent breakthroughs in therapy, CVD remains the leading cause of death in the developed world, accounting for about one million deaths annually in the United States alone. About 17.7 million people died from CVDs globally in 2015, representing 31% of all deaths in the world [1]. By 2030, over 23.6 million people will have died from CVDs, primarily heart disease and stroke. These are expected to be the primary causes of death for the foreseeable future [2]. Traditional CVD risk factors aren’t responsible for all deaths. Environmental, nutritional, and lifestyle factors appear to be crucial in explaining the dramatic recent changes in the prevalence, with the potential of widespread public health implications [3]. Recent research has shown that heavy metal exposure is related to an increased risk of cardiovascular diseases [1, 4, 5].

Heavy metals enter the human body through multiple routes including food, drinking water, and breathing. Heavy metals include toxic metals such as arsenic (As), cadmium (Cd), lead (Pb), and mercury (Hg), as well as vital trace elements such as chromium (Cr), cobalt (Co), copper (Cu), magnesium (Mg), manganese (Mn), nickel (Ni), selenium (Se) and zinc (Zn) [6]. Multiple heavy metal exposures can have additive, synergistic, antagonistic, or other effects on human health [7, 8], however, most studies of heavy metals focus on single metal [9]. In addition, many earlier studies on heavy metals’ negative effects tended to focus on occupational exposure alone [5, 10, 11]. Heavy metal workers are exposed to higher amounts, while the general public in the United States is exposed to lower levels [12]. Low dosages of heavy metals produce epidemiological outcomes that are more in line with actual ambient exposure levels, and their exposure has been found in studies to be hazardous to the population [13]. Biologically active metals do, therefore, have a role across a range of physiological and pathological processes [14].

Evidence of the involvement of environmental exposure to heavy metals in CVD risk has quickly increased during the past two decades. Recent research points to evidence associating heavy metal exposure in the environment with an amplified risk for diabetes and hypertension, two major risk factors for CVD [3]. Higher amounts of barium in drinking water have been linked to increased cardiovascular mortality [15]. A Spanish study found that urine Cu, Zn, antimony (Sb), Cd, Cr, and vanadium (V) levels were all independently related to an elevated risk of cardiovascular diseases. Urine metals were similarly linked to an increased risk of cardiovascular diseases, with Cd and Sb being the most significant components [16]. In addition, heavy metals were found to interact with other diseases leading to CVD. An interaction between blood Cd and chronic bronchitis was reported to be associated with myocardial infarction, and interaction between blood Pb level and chronic obstructive pulmonary disease (COPD) was associated with a heart attack or stroke [1, 5]. However, most of these studies only examined the exposure effects of one heavy metal at a time.

Estimating the health effects of multi-pollutant exposures is of critical concern in environmental epidemiology and to regulatory agencies as humans are frequently exposed to many metals throughout their lifetimes. Using the weighted quantile sum (WQS) model, Duan et al. [8] investigated the correlations between a heavy metal combination and the risks of all-cause, CVD-related, and cancer-related death. Methods have been proposed for joint modelling of the data. Dunson [18] presented a new class of latent variables for grouping mixed outcome data. Nonetheless, several issues must be addressed in order to accurately quantify the health impacts of these multi-pollutant combinations. While current techniques for investigating mixtures [19] address some of these difficulties, they also have significant drawbacks. Bobb et al. [20] developed Bayesian kernel machine regression (BKMR) as a novel way to study mixtures, in which the health outcome is regressed on a flexible function of the mixture’s components (e.g., air pollution or hazardous waste) that is described using a kernel function. A unique hierarchical variable selection strategy is used in high-dimensional situations to find essential mixture components and accounts for the associated structure of the mixture. This BKMR model is used in the current study to identify blood and urine heavy metals and heavy metal mixtures that may be associated with heart attacks.

## Materials and Methods

### Study population

The data for this study comes from the National Health and Nutrition Examination Survey (NHANES) [21] covering the period from 2011 through 2016. It has been an ongoing national, population-based cross-sectional survey of the US population since the 1980s. We used Demographics Data, Medical Conditions, Laboratory Data, and Questionnaire Data in this analysis. Except for the levels of heavy metal, all variables were self-reported. We only considered participants who were 20 years or older. We also excluded subjects with missing data. The final sample contained 2972 participants.

### Measurement of diseases

The survey question “Has a doctor or other health professional ever told you that you had a heart attack?” from the Medical Conditions data set was used to determine the study’s main outcome: the occurrence of a heart attack. Some frequent comorbidities, such as high blood cholesterol, high blood pressure, poor kidney function, diabetes, and asthma, were also included as covariates [4, 5, 8]. These variables were determined by the question “Has a doctor or other health professional ever told you that you had high blood pressure/high cholesterol/weak or failing kidney/diabetes/asthma?”

### Measurement of Heavy Metals

Blood lead, cadmium, mercury, and manganese level were all extracted from a lab data set called the lead, cadmium, total mercury, selenium, and manganese-blood. The Metals – Urine dataset of Laboratory Data was used to collect urine cobalt and barium information. More details, information measurement procedures, and quality control processes can be found on NHANES 2011–2012, 2013–2014, and 2015–2016 Data Documentation [21].

### Measurement of smoking and alcohol intake

The Smoking-Cigarette Usage dataset was utilized to determine smoking status with the queries “Smoked at least 100 cigarettes in your life?” and “Do you now smoke cigarettes?”. Smokers were recoded as never smokers, former smokers, and current smokers. Alcohol was a continuous variable that showed the average number of alcoholic beverages drank per week by people in the preceding year.

### Measurement of other covariates

Gender, Age, Marital Status, Household Income, Race, Educational Level, and Body Mass Index (BMI) were obtained from the NHANES Demographic, Examination, and Questionnaire data sets [21]. Based on earlier research [1, 5, 8] cut points were appropriately chosen.

### Statistical Analysis

R statistical software, version 4.1.1 [22], was used for all analyses. To account for the complex, multistage survey design, a SURVEYLOGISTIC procedure was applied. We incorporated the weight, stratum, and cluster variables from NHANES data in the procedure [22]. Using survey logistic techniques, these three variables were included in the univariate models. to account for design features. The goal of the univariate logistic model investigation was to include multiple metals in the analysis to create models that more accurately represent real-life exposures. The chi-square test was performed to examine the relationship between heart attack history and categorical variables. The t-test was performed to compare the equality of means in continuous variables between groups with and without a heart attack. Pearson correlation test was used to find out the correlation between the metals. Adjusted Odds Ratios and 95% credible intervals (CI) were obtained from the univariate logistic regression and Bayesian kernel machine regression analysis respectively.

The ‘BKMR’ package [23] that implements Bayesian Kernel Machine Regression in R was used to see if there were any significant associations between the mixture of heavy metal levels and heart attack status. The BKMR model, a non-parametric Bayesian variable selection framework was used to evaluate the mixture effect of metals on heart attack. BKMR combines Bayesian and statistical learning methods to regress an exposure-response function iteratively by a Gaussian kernel function. BKMR can identify nonlinear and non-additive relationships within metals. In the current study, the outcome of interest (Y = 1) is heart attack (is binary), and the exposure variables z are blood lead, cadmium, mercury, and manganese and urine cobalt and barium, we used the following probit BKMR model.

$$\Phi^{-1}\left(\mu_{\text{i}}\right)=\text{h}\;\left({\text{z}}_{\text{i}1},{\text{z}}_{\text{i}2},\ldots ,{\text{z}}_{\text{iM}}\right)+{\text{x}}_{\text{i}}^{\prime }\beta$$

where **Φ** is the cumulative distribution function (CDF) for the standard normal distribution (**Φ**^−1^ is the probit link function) and ^ is the probability that Y_i_ equals 1. The h (·) is an exposure-response function that flexibly models the relationship between the exposures to multiple metals z_1_…z_M_, and the probit of the probability of a heart attack (Y=1). The x is a vector of non-exposure covariates with a linear or non-linear relationship with the outcome, and p is a vector of respective coefficients of x [20, 23, 24].

Under BKMR, the kernel function used to represent h has several options. In this section, we concentrate on the Gaussian kernel, which captures a wide range of underlying functional forms for h and can be expressed as

$$K\;\left(z,z^{\prime }\right)=\text{exp}\;\left\{-\sum_{m=1}^{M}r_{m}{\left(z_{m}-{z^{\prime }}_{m}\right)}^{2}\right\}$$

In this case, both z and z^1^ represent a vector of exposure variables for two different individuals. In the present context, M is the number of metals, and *r*_*m*_ ≥ 0is the tuning parameter for the smoothness of h. With this kernel function, it is assumed that similar exposure profiles will have similar health effects. In the current study, this means two individuals with similar blood and urine heavy metal exposures will have a similar risk of a heart attack. To estimate h(z) at a certain exposure vector z, the posterior distribution of h is assumed to be normally distributed, with a posterior mean *μ*_*h*_ (*θ*)and variance *V*_*h*_ (*θ*), which depends on the model parameters denoted by *θ* [20, 23].

The probit model above can be expressed using a latent normal random variable formulation as follows:

$${Y^{*}}_{i}=h\;\left(z_{i1},z_{i2},\ldots ,z_{iM}\right)+{x^{\prime }}_{i}\beta +e_{i}$$

where *e*_*i*_ assumes standard normal distribution and

$$Y_{i}=I\;\left({Y^{*}}_{i}>0\right)=\{\begin{array}{l} 1\;if{Y^{*}}_{i}>0\\ 0\text{otherwise} \end{array}$$

The Markov chain Monte Carlo (MCMC) algorithm is implemented to fit the BKMR model and allows for customization of the model with options such as continuous or binary outcomes, random or non-random intercepts, component wise variable selection, and hierarchical variable selection [20, 23, 24]. The model fit for the current study used binary outcomes, non-random intercepts, component wise variable selection, and the MCMC algorithm. The BKMR model is not able to accommodate sample weights yet, and thus we used unweighted estimation [25].

The R software and the required packages can be obtained from the CRAN website [22]. More details regarding the ‘bkmr’ package code and usage can be found in Bobb [23] and Bobb et al [24].

## Results

Table 1 shows the general features of our study population. Of the 2972 participants included in the study, 89 (3%) suffered from a heart attack. The prevalence of heart attack was higher in groups older than 60, men, who had high blood pressure, and high blood cholesterol and suffered from other comorbidities such as diabetes, asthma, and weak kidney functions. Smokers (including ex-smokers and current smokers) and heavy drinkers were more likely to have a heart attack (p-value <0.001). There were significantly different mean levels of heavy metals in the blood and urine of the heart attack group compared to the non-heart attack group (p-value <0.001). Those who had a heart attack exhibited higher mean levels of cadmium and lead in the blood as well as higher mean levels of cadmium, cobalt, and tin in the urine compared to those who did not report a heart attack. Conversely, the heart attack group had lower mean levels of mercury, manganese, and selenium in the blood and manganese, barium, tungsten, and strontium in the urine.

**Figure 1:**
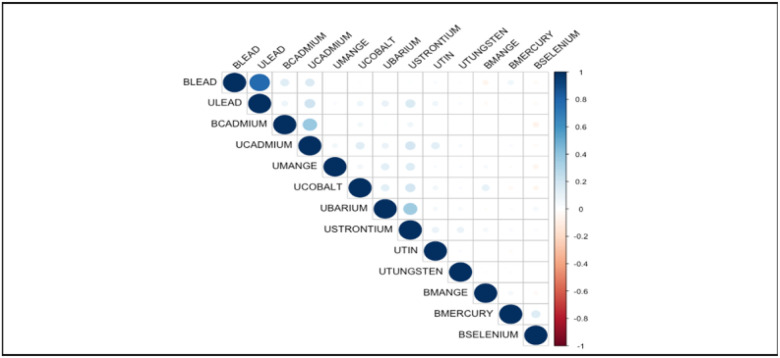
Correlation between Blood and Urine Metals

At the beginning of our analysis, we intended to include all metal exposures in our dataset in order to evaluate their associations with heart attack. However, during our initial assessment, we found that blood and urine lead were significantly associated (Pearson correlation coefficient of 0.78) as shown in figure 1. As a result of the potential of multicollinearity, we decided not to include urine lead in our study. Multicollinearity occurs when independent variables in a regression model have a strong correlation, which can lead to unstable or inaccurate estimates of their individual effects on the outcome variable. In this case, including both blood and urine lead in the analysis could have made it difficult to determine the independent effects of each exposure on the health outcome, as they would be highly correlated with each other. We aimed to reduce the risk of multicollinearity and obtain more reliable estimates of the associations between the remaining metal exposures and the heart attack by excluding urine lead from the analysis [1,5,15,16].

The results of the univariate survey logistic regression analysis are presented in table 2. Looking at individual heavy metal exposures, we observed that the odds of heart attack increased by about 10% in patients for every 1 μ/L increase in the average level of blood lead (OR = 1.014, CI = [1.005 – 1.022]) and by 91% for every 1 μ/L increase in the average level of blood cadmium (OR = 1.911, CI = [1.413 – 2.584). Several patient characteristics were associated with increased odds of heart attack. Males had 2.46 times the odds of heart attack compared to females (OR = 2.463, CI = [1.349 – 4.496]). Individuals in the 40–59 year (OR = 5.095, CI = [1.349 – 19.253]), 60–74 year (OR = 23.839, CI = [8.891– 72.022]), and ≥75 year (OR = 36.295, CI = [10.575 – 12.457]) age groups also had significantly greater odds of heart attack compared to those in the 20–39 year age group. Compared to those who were married, patients who were widowed (OR = 3.039, CI = [1.578, 5.852]) had greater odds of a heart attack. Patients with diabetes (OR = 8.687, CI = [4.389 – 17.195]), high blood pressure (OR = 6.08, CI = [3.659, 10.101]), and high blood cholesterol (OR = 6.686, CI = [3.641, 12.279]) had increased odds of having a heart attack. Current and former smokers had higher odds of heart attack compared to those who never smoked (OR = 2.797, CI = [1.440, 5.436]; OR = 1.929, CI = [0.932, 3.994] respectively). By contrast, other patient characteristics were associated with lower odds of a heart attack. For example, Mexican American (OR = 0.36, CI = [0.135, 0.959]) patients had lower odds of heart attack compared to non-Hispanic White patients. Patients whose household income was greater than or equal to $20,000 also had lower odds of heart attack than patients whose household income was less than $20,000 (OR = 0.475, CI = [0.314, 0.718]). Similarly, patients with more than a high school education (OR = 0.383, CI = [0.180, 0.810]) had lower odds of having a heart attack than patients with less than high school education.

Using Bayesian Kernel Machine Regression implemented in the R ‘bkmr package [23], which is a statistical approach for estimating the joint health effects of multiple concurrent exposures, we investigated the mixture effect of heavy metals on heart attack.

We fitted the BKMR model to evaluate how the joint effect of metal exposures impacts heart attack risk. Table 3 summarizes the posterior inclusion probability (PIP) derived from the model, which measures variable importance. PIP is a ranking measure that indicates how strongly the data supports the inclusion of a variable in the regression. For example, a PIP value of 0.5104 for Lead in the Blood exposure column indicates a 51.04% possibility that Lead exposure is associated with an increased risk of heart attack. Similarly, the PIP value of 0.7872 for Cadmium in the Urine exposure column indicates that there is a 78.72% possibility that Cadmium exposure is associated to an increased risk of heart attack.

Next, we estimated the Cumulative effects of metal mixtures on heart attack as the mixture exposure changes in the index (or linear predictor) h(z) (table 4). Changes in h are the results of combined changes in any components of the metal mixture. Table 4 summarizes the cumulative effect of the mixture exposure h(z) on the risk of a heart attack in comparison with that at the median level of h(z). The “Fraction of Risk Change (in Probit)” column indicates the change in risk relative to the median exposure level, measured in probits (a unit of measurement for standard deviations). The “Standard Deviation” column indicates the uncertainty or variability of the risk estimate.

At the median exposure level (quantile 0.5), the risk change is 0.000, which means that there is no effect of metal mixtures on relative risk at this level of exposure. At the lower exposure quantiles (0.25, 0.3, and 0.4), the risk change is negative, indicating a decrease in risk with increasing metal mixture exposure. For example, the effect for the 0.25 quantile of exposure is −0.0030, indicating a small decrease in risk with increasing exposure to metal mixtures. The corresponding standard deviation of 0.0618 suggests that this effect is not statistically significant. At the higher exposure quantiles (0.6, 0.7, and 0.75), the risk change is positive, indicating an increase in risk with increasing metal mixture exposure. The effect for the 0.75 quantile of exposure is 0.0285, indicating an increase in risk with increasing exposure to metal mixtures. However, the standard deviation is relatively larger for these quantiles, which means that the risk estimates are less certain. Overall, the results suggest that the effect of metal mixtures on relative risk depends on the level of exposure, with lower exposure levels being associated with a decrease in risk and higher exposure levels being associated with an increase in risk.

**Figure 2:**
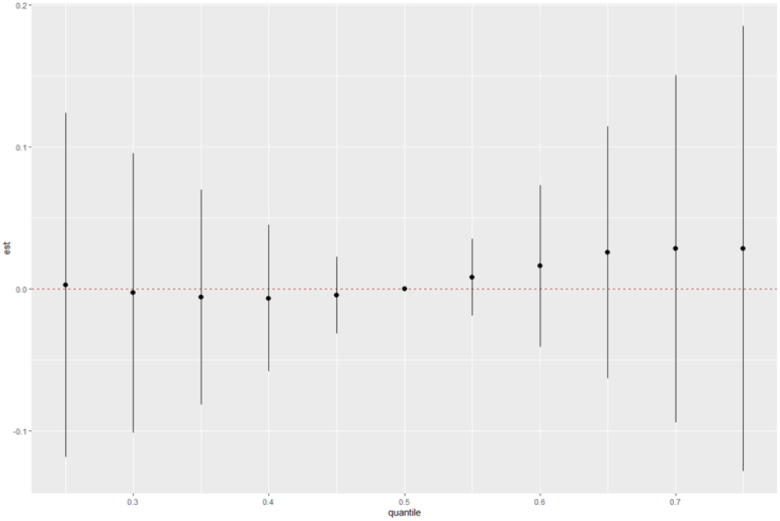
Overall Risk Summaries

Figure 2 displays the change in heart attack risks at different levels of the mixture exposure to the six metals when compared with each of the three metals at their median value (i.e., 50^th^ percentile). The risk of having a heart attack showed an increase when all the metals were at their 60^th^, 70^th^, and 75^th^ percentile compared to their 50^th^ percentile, indicating a positive association. Overall, we found a nonlinear relationship between the risk of heart attack and the mixture of heavy metals.

Table 5 presents the estimated risk differences and their respective 95% confidence intervals for each metal exposure at different fixed quantiles of the remaining exposures. A positive risk difference indicates an increased risk of heart attack as metal exposure increases, whereas a negative difference indicates a decreased risk.

**Figure 3:**
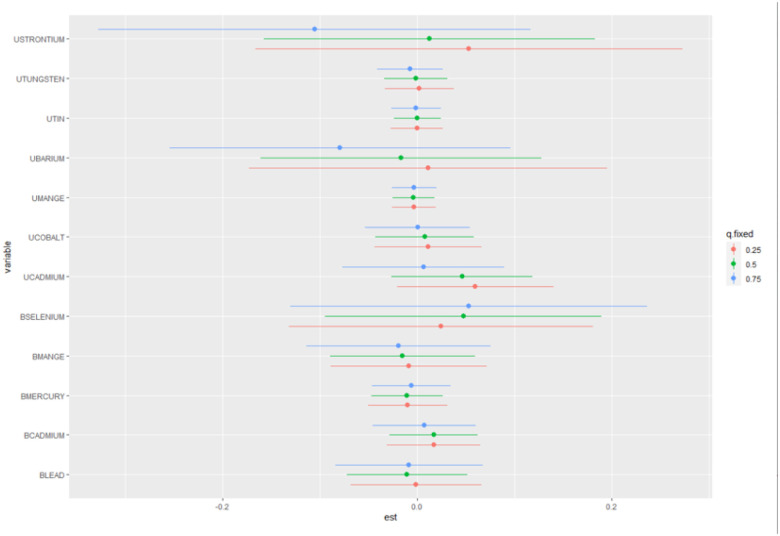
Individual Exposures Effect on the Heart Attack

For blood cadmium, there was a small positive risk difference at the 25th, 50th, and 75th quantiles, but the credible intervals included zero, indicating that the estimated risks were not statistically significant. The estimated risk differences for blood lead, blood manganese, blood mercury, and urine manganese were negative but not statistically significant at any quantile, as the credible intervals included zero. The estimated risk differences for urine barium were positive at the 25th quantile but negative at the 50th and 75th quantiles, however there was no statistically significant association between urine barium level and heart attack. For blood selenium, urine cadmium, urine cobalt and urine strontium the estimated risk were positive at all quantiles, but the credible intervals included zero, indicating that the estimated changes were not statistically significant. Figure 3 illustrates a visual representation of these numerical summaries, making it easier to identify the relative contributions of individual exposures to the heart attack.

**Figure 4:**
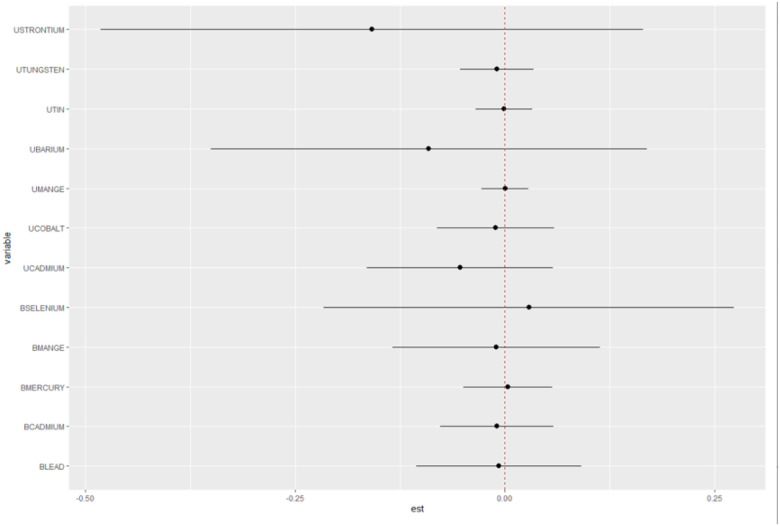
Interaction Effect between the Metals

We also compared the health risks of each metal exposure when all the other exposures are fixed to their 75th percentile to when all of the other exposures are fixed to their 25th percentile. The analysis results showed no significant evidence of interactions between metal exposures and the outcome. The credible intervals for all metal exposures included zero, indicating that the estimated interaction effects were not statistically significant. These findings are presented in Figure 4, which provides a visual representation of the interaction effects between the metal exposures.

## Discussion

The research investigates the association between heavy metal exposure and the likelihood of having a heart attack. Males, people over 60 years old, those with high blood pressure and cholesterol, diabetes, asthma, poor kidney function, smokers, and heavy drinkers have an increased risk of having a heart attack. The analyses show that people who have had a heart attack have greater blood levels of cadmium and lead, as well as higher urine levels of cadmium, cobalt, and tin. Males, patients over the age of 40, and those with diabetes, high blood pressure, and high blood cholesterol are more likely to suffer a heart attack, according to the univariate survey logistic regression analysis. The study also investigates the combined effect of heavy metals on heart attack using Bayesian Kernel Machine Regression. The findings suggest that exposure to lead, cadmium, and tin is associated to an increased risk of heart attack.

Most of the past research has looked at the association between individual heavy metals and CVD. To our knowledge, this is the first study to investigate the association between the mixture of heavy metals from both blood and urine and the prevalence of heart attacks. Results of this study confirmed the primary hypothesis that exposure to a mixture of blood and urine heavy metals was significantly associated with a heart attack.

Previous research has identified links between heavy metals exposure and heart attacks. Generally, just one single metal was included in these experiments, making the results easy to understand. However, to better represent real-life exposures, we must include a variety of heavy metal exposures as well as their complicated, nonlinear relationships [25]. Ignoring the combined impact of other metals may result in misleading positive or false negative results [26]. Nonetheless, caution must also be exercised when including all the metals of interest in a single multivariate regression model because this may lead to result distortion [27, 28]. The approach of the multivariate survey logistic model presented here aimed to mindfully include multiple metals in the analysis to build models that more accurately represented real-life exposures.

The BKMR model was developed to analyze the effects of exposure mixtures on health. Our findings contribute to the knowledge of how a mixture of heavy metals influences the risk of heart attack. This approach allows for the examination of the overall mixture effect as well as the impact of each mixture component in the context of the overall joint exposure. By applying hierarchical variable selection, the approach may be able to identify the most critical windows of susceptibility while allowing for highly correlated exposures. Finally, when estimating a high-dimensional collection of exposures, a Bayesian method is used to account for uncertainty. Using BKMR in this study population we predicted a joint effect of blood and urine heavy metals on the heart attack that showed a significant decline when all metals were at the lower percentile (e.g., 25th percentile) and an increase when all metals were at the higher percentile (e.g., 70th percentile) compared to the 50th percentile increment, with the highest risk noted at the 60th percentile. We also examined the interaction effect of social stressors (gender, age, household income, and education status) and metal mixtures, but no significant interactions were found (data not shown).

A population-based study from Spain [16] found that increased levels of urine Cu, Zn, Sb, Cd, Cr, and V individually and as a mixture was associated with increased risk of any fatal or non-fatal cardiovascular incidents that collectively fall under the International Classification of Diseases 10th Revision (ICD-10) codes I00-I78, and BKMR analysis indicated that Cd and Sb were the main drivers of the association when considering the metals as a mixture. Another study of NHANES data from 1999–2014 found that heavy metal mixtures measured in blood and urine increased the odds of CVD-related death [8]. While studies investigating the effect of metal mixtures on cardiovascular disease outcomes are limited, many studies have highlighted the effects of individual heavy metals detected in blood and urine on CVD. For instance, one study found that higher levels of blood or urine cadmium increased the risk of stroke and heart failure [29]. Two studies among Pakistani myocardial infarction patients found that these patients had higher levels of blood mercury and urine manganese compared to healthy age-matched reference patients [30, 31]. Another study of NHANES data from 1999–2006 found that higher levels of blood cadmium or cobalt were associated with higher odds of cardiovascular disease [4]. While many studies indicate an association between heavy metal exposure and cardiovascular disease, the mechanisms driving this association are still unclear. Research suggests that exposure to heavy metals or metal mixtures lead to increased oxidative stress, inflammation, and cardiac cell death [32, 33], a biological process that could explain the increased risk of cardiovascular disease associated with these exposures.

Our research provided helpful insights, but it is important to acknowledge its limitations. We did not collect samples prior to the onset of the diseases, making it difficult to identify the exact time of exposure. However, we made use of convenient samples that were available to us and attempted to extract as much information as possible from them. Although these samples may not be a direct indicator of prior exposure levels, we believe that the measurements we obtained reflect a relatively stable exposure situation that is not likely to have changed significantly. Another limitation of the study is that because it is cross-sectional, it is difficult to make causal assumptions. Another disadvantage is the likelihood of misclassification bias due to self-reporting (e.g., the outcome variable, myocardial infarction), a method that is vulnerable to variable degrees of inaccuracy Despite this, there are several beneficial aspects to the study. This is a population-based study that used data from the National Health and Nutrition Examination Survey (NHANES), which obtains high-quality data while adhering to strict criteria requirements to minimize errors. One other limitation of our study was the number of iterations was low due to the lack of computing power. Nonetheless, BKMR has the advantage of not only addressing the mixture effect but also of being able to extract the contributions of each component, with the caveat that these contributions are in the context of joint exposure at the exposure levels reported in the cohort. Finally, by using the BKMR method, we were able to overcome significant drawbacks of conventional analytic pathways, such as single metal effect estimate, model misspecification, and increased false discovery when fitting multiple regression models.

## Conclusions

In conclusion, exposure to the mixture of heavy metals considered in this study was associated with an increased risk of a heart attack. Exposed adults aged 20 and above had a greater likelihood of heart attack related to levels of a heavy metal combination. When evaluating the entire mixture, lead, and selenium in the blood and cadmium, manganese, tin and strontium in the urine were found to be the most significant exposures associated with heart attacks.

The BKMR model presented in this study can be used to explore new types of exposure in future studies, with the potential of yielding enhanced knowledge of how the environment as a whole influences’ health and disease in different population settings. While the current study found a significant association between exposure to mixtures of heavy metals and heart attack, additional studies using larger cohorts are needed to estimate the effects of heavy metal mixtures on a heart attack at exposure levels that are relevant for general populations. We also suggest using different approaches and interpreting their results together to draw more robust conclusions.

## Figures and Tables

**Table 1.** Baseline Characteristics from NHANES 2011–2016, of Adults More Than 20 Years Old

Characteristics	Heart Attack = No (N=2883)		Heart Attack = Yes (N=89)	Overall (N=2972)	p-value
**Gender**					**0.001**
Female	1357 (47.1%)		26 (29.2%)	1383 (46.5%)	
Male	1526 (52.9%)		63 (70.8%)	1589 (53.5%)	
**Age**					**<0.001**
20–39	1191 (41.3%)		5 (5.6%)	1196 (40.2%)	
40–59	982 (34.1%)		19 (21.3%)	1001 (33.7%)	
60–74	561 (19.5%)		43 (48.3%)	604 (20.3%)	
≥ 75	149 (5.2%)		22 (24.7%)	171 (5.8%)	
**Marital Status**					**<0.001**
Never Married	660 (22.9%)		12 (13.4%)	672 (22.6%)	
Married	1428 (49.5%)		44 (49.4%)	1472 (49.5%)	
Widowed	135 (4.7%)		13 (14.6%)	148 (5.0%)	
Divorced	303 (10.5%)		11 (12.4%)	314 (10.6%)	
Separated	78 (2.7%)		5 (5.6%)	83 (2.8%)	
Living with Partner	279 (9.7%)		4 (4.5%)	283 (9.5%)	
**Household Income**					**<0.001**
< USD 20,000	490 (17.0%)		33 (37.1%)	523 (17.6%)	
≥ USD 20,000	2393 (83.0%)		56 (62.9%)	2449 (82.4%)	
**Race**					0.079
Mexican American	371 (12.9%)		7 (7.9%)	378 (12.7%)	
Other Hispanic	281 (9.7%)		9 (10.1%)	290 (9.8%)	
Non-Hispanic White	1234 (42.8%)		45 (50.6%)	1279 (43.0%)	
Non-Hispanic Black	603 (20.9%)		23 (25.8%)	626 (21.1%)	
Other Race	394 (13.7%)		5 (5.6%)	399 (13.4%)	
**Education**					**0.003**
< High School	463 (16.1%)		22 (24.7%)	485 (16.3%)	
High School	596 (20.7%)		27 (30.3%)	623 (21.0%)	
> High school	1824 (63.2%)		40 (44.9%)	1864 (62.7%)	
**High Blood Pressure**					**<0.001**
No	1956 (67.8%)		25 (28.1%)	1981 (66.7%)	
Yes	927 (32.2%)		64 (71.9%)	991 (33.3%)	
**High Cholesterol**					**<0.001**
No	1927 (66.8%)		30 (33.7%)	1957 (65.8%)	
Yes	956 (33.2%)		59 (66.3%)	1015 (34.2%)	
**Diabetes**					
No	2533 (87.9%)	53 (59.6%)		2586 (87.0%)	**<0.001**
Yes	280 (9.7%)	35 (39.3%)		315 (10.6%)	
Borderline	70 (2.4%)	1 (1.1%)		71 (2.4%)	
**Kidney Conditions**					**<0.001**
No	2815 (97.6%)	81 (91.0%)		2896 (97.4%)	
Yes	68 (2.4%)	8 (9.0%)		76 (2.6%)	
**BMI**					0.853
< 18.5	42 (1.5%)	1 (1.1%)		43 (1.4%)	
18.5 – 24.9	809 (28.1%)	25 (28.1%)		834 (28.1%)	
25 – 29.9	934 (32.4%)	26 (29.2%)		960 (32.3%)	
30+	1098 (38.1%)	37 (41.6%)		1135 (38.2%)	
**Alcohol Consumption**					**<0.001**
Mean (SD)	3.02 (18.7)	2.76 (2.60)		3.02 (18.4)	
**Smoking Status**					**<0.001**
Never	1533 (53.2%)	26 (29.2%)		1559 (52.5%)	
Current	632 (21.9%)	32 (36.0%)		664 (22.3%)	
Former	718 (24.9%)	31 (34.8%)		749 (25.2%)	
**Asthma**					**<0.001**
No	2426 (84.1%)	63 (70.8%)		2489 (83.7%)	
Yes	457 (15.9%)	26 (29.2%)		483 (16.3%)	
**Blood lead (ug/L)**					**<0.001**
Mean (SD)	13.5 (14.9)	21.1 (26.7)		13.7 (15.4)	
Median [Min, Max]	9.90 [1.10, 337]	15.1 [4.00, 246]		10.1 [1.10, 337]	
**Blood cadmium (ug/L)**					**<0.001**
Mean (SD)	0.480 (0.555)	0.741 (0.728)		0.487 (0.563)	
Median [Min, Max]	0.290 [0.070, 7.23]	0.510 [0.070, 4.00]		0.300 [0.070, 7.23]	
**Blood mercury (ug/L)**					**<0.001**
Mean (SD)	1.62 (2.64)	1.38 (1.56)		1.61 (2.62)	
Median [Min, Max]	0.860 [0.110, 50.8]	0.940 [0.200, 9.12]		0.860 [0.110, 50.8]	
**Blood manganese (ug/L)**					**<0.001**
Mean (SD)	9.78 (3.60)	9.53 (3.29)		9.77 (3.59)	
Median [Min, Max]	9.17 [1.88, 56.6]	8.87 [4.55, 23.5]		9.16 [1.88, 56.6]	
**Blood selenium (ug/L)**					**<0.001**
Mean (SD)	196 (24.2)	191 (23.5)		196 (24.2)	
Median [Min, Max]	195 [106, 391]	192 [120, 260]		195 [106, 391]	
**Urine cadmium (ug/L)**					**<0.001**
Mean (SD)	0.315 (0.431)	0.500 (0.399)		0.320 (0.431)	
Median [Min, Max]	0.181 [0.025, 6.94]	0.386 [0.041, 1.93]		0.186 [0.025, 6.94]	
**Urine Lead (ug/L)**					**<0.001**
Mean (SD)	5.52 (10.6)	6.70 (6.44)		5.56 (10.5)	
Median [Min, Max]	3.60 [0.200, 350]	4.40 [0.500, 39.4]		3.60 [0.200, 350]	
**Urine cobalt (ug/L)**					**<0.001**
Mean (SD)	0.503 (0.897)	0.680 (1.19)		0.508 (0.907)	
Median [Min, Max]	0.354 [0.016, 33.7]	0.358 [0.032, 9.91]		0.355 [0.016, 33.7]	
**Urine manganese (ug/L)**					**<0.001**
Mean (SD)	0.152 (0.490)	0.149 (0.248)		0.151 (0.485)	
Median [Min, Max]	0.0920 [0.057, 18.2]	0.0920 [0.057, 2.38]		0.0920 [0.057, 18.2]	
**Urine barium (ug/L)**					**<0.001**
Mean (SD)	1.78 (3.06)	1.53 (1.65)		1.77 (3.03)	
Median [Min, Max]	1.08 [0.042, 87.4]	0.900 [0.060, 9.06]		1.08 [0.042, 87.4]	
**Urine tin (ug/L)**					**<0.001**
Mean (SD)	1.23 (4.02)	2.51 (9.76)		1.27 (4.31)	
Median [Min, Max]	0.440 [0.064, 89.9]	0.770 [0.064, 91.0]		0.450 [0.064, 91.0]	
**Urine tungsten (ug/L)**					**<0.001**
Mean (SD)	0.121 (0.645)	0.115 (0.207)		0.121 (0.637)	
Median [Min, Max]	0.0580 [0.013, 32.9]	0.0770 [0.013, 1.85]		0.0590 [0.013, 32.9]	
**Urine strontium (ug/L)**					**<0.001**
Mean (SD)	120 (120)	108 (95.9)		119 (119)	
Median [Min, Max]	90.2 [1.77, 3220]	78.3 [5.6, 542]		89.8 [1.77, 3220]	

**Table 2:** Odds Ratio of Univariate Model

Characteristics	Odds Ratio (OR)	95% CI
**Blood lead level**		
Mean	1.014	[1.005, 1.022]
**Blood cadmium level**		
Mean	1.911	[1.413, 2.584]
**Blood mercury level**		
Mean	0.999	[0.926, 1.077]
**Blood manganese level**		
Mean	1.019	[0.936, 1.109]
**Blood selenium level**		
Mean	0.992	[0.979, 1.006]
**Urine cadmium level**		
Mean	1.892	[1.298, 2.758]
**Urine cobalt level**		
Mean	1.284	[0.892, 1.848]
**Urine manganese level**		[0.230, 2.228]
Mean	0.715	
**Urine barium level**		
Mean	1.012	[0.955, 1.073]
**Urine tin level**		
Mean	1.034	[1.010, 1.058]
**Urine tungsten level**		
Mean	1.016	[0.882, 1.169]
**Urine strontium level**		
Mean	0.023	[0.999, 1.002]
**Gender**		
Female	reference	
Male	2.463	[1.349, 4.496]
**Age**		
20 –39	reference	
40 – 59	5.095	[1.349, 19.253]
60 – 74	23.839	[8.891, 72.022]
≥ 75	36.295	[10.575, 12.457]
**Marital Status**		
Married	reference	
Never Married	0.502	[0.206, 1.224]
Widowed	3.039	[1.578, 5.852]
Divorced	0.563	[0.236, 1.343]
Separated	3.134	[0.912, 10.771]
Living with Partner	0.526	[0.117, 2.364]
**Household Income**		
< USD 20,000	reference	
≥ USD 20,000	0.475	[0.314, 0.718]
**Race**		
Non-Hispanic White		
Other Hispanic	reference	
Mexican American	0.643	[0.282, 1.466]
Non-Hispanic Black	0.360	[0.135, 0.959]
Other Race	1.282	[0.738, 2.228]
	0.716	[0.161, 3.175]
**Education**		
< High School	reference	
High School	0.557	[0.263, 1.179]
> High school	0.383	[0.180, 0.810]
**Diabetes**		
No	reference	
Yes	8.687	[4.389, 17.195]
Borderline	0.159	[0.020, 1.269]
**High Blood Pressure**		
No	reference	
Yes	6.080	[3.659, 10.101]
**High Cholesterol**		
No	reference	
Yes	6.686	[3.641, 12.279]
**BMI**		
< 18.5	reference	
18.5 – 24.9	1.405	[0.166, 11.879]
25 – 29.9	1.571	[0.182, 13.561]
30+	1.725	[0.206, 14.483]
**Alcohol Consumption**	1.000	[0.996, 1.005]
Mean		
**Smoking Status**		
Never	reference	
Current	2.797	[1.440, 5.436]
Former	1.929	[0.932, 3.994]
**Kidney Conditions**		
No	reference	
Yes	2.584	[1.013, 6.591]
**Asthma**		
No	reference	
Yes	2.194	[1.160, 4.147]

**Table 3:** Posterior Inclusion Probability (PIP)

Exposure	PIP
**Blood**	
Lead	0.5104
Cadmium	0.4088
Mercury	0.4376
Manganese	0.3280
Selenium	0.5200
**Urine**	
Cadmium	0.7872
Cobalt	0.4304
Manganese	0.5272
Barium	0.4880
Tin	0.5208
Tungsten	0.4304
Strontium	0.5504

**Table 4:** Total effects of metal mixtures relative risk at median exposure Effect

Quantile	Fraction of Risk Change (in Probit)	Standard Deviation
0.25	−0.0030	0.0618
0.3	−0.0027	0.0502
0.4	−0.0065	0.0263
0.5	0.000	0.000
0.6	0.0162	0.0291
0.7	0.0283	0.0623
0.75	0.0285	0.0800

**Table 5:** Single Exposure Effects

Metal Exposure	Fixed Quantile of Remaining Exposures	Estimated Risk Difference (change)	95% Credible Interval
Blood Cadmium	0.25	0.0168	[−0.0315, 0.0621]
	0.5	0.0168	[−0.0286, 0.0622]
	0.75	0.0072	[−0.0457, 0.0602]
Blood Lead	0.25	−0.0012	[−0.0684, 0.0660]
	0.5	−0.0104	[−0.0722, 0.0514]
	0.75	−0.0085	[−0.0841, 0.0672]
Blood Manganese	0.25	−0.0089	[−0.089, 0.0711]
	0.5	−0.0150	[−0.0894, 0.0594]
	0.75	−0.0194	[−0.1140, 0.0752]
Blood Mercury	0.25	−0.0097	[−0.0503, 0.0309]
	0.5	−0.0104	[−0.0472, 0.0264]
	0.75	−0.0062	[−0.0465, 0.0341]
Blood Selenium	0.25	0.0246	[−0.1316, 0.1808]
	0.5	0.0474	[−0.0946, 0.1894]
	0.75	0.0530	[−0.1304, 0.2364]
Urine Barium	0.25	0.0114	[−0.1726, 0.1954]
	0.5	−0.0168	[−0.1611, 0.1274]
	0.75	−0.0793	[−0.2546, 0.0961]
Urine Cadmium	0.25	0.0598	[−0.0206, 0.1402]
	0.50	0.0462	[−0.0263, 0.1187]
	0.75	0.0063	[−0.0766, 0.0891]
Urine Cobalt	0.25	0.0112	[−0.0437, 0.0662]
	0.5	0.0076	[−0.0433, 0.0585]
	0.75	0.0003	[−0.0538, 0.0544]
Urine Manganese	0.25	−0.0036	[−0.0261, 0.0190]
	0.5	−0.0039	[−0.0254, 0.0176]
	0.75	−0.0033	[−0.0262, 0.0195]
Urine Strontium	0.25	0.0532	[−0.1665, 0.2728]
	0.5	0.0126	[−0.1576, 0.1827]
	0.75	−0.1055	[−0.3275, 0.1165]
Urine Tin	0.25	−0.0003	[−0.0271, 0.0264]
	0.5	0.0001	[−0.0242, 0.0243]
	0.75	−0.0013	[−0.0268, 0.0243]
Urine Tungsten	0.25	0.0022	[−0.0333, 0.0377]
	0.5	−0.0016	[−0.034, 0.0307]
	0.75	−0.0075	[−0.0413, 0.0263]

## Data Availability

Data will be available upon contacting corresponding author.
